# Mir-370-3p Impairs Glioblastoma Stem-Like Cell Malignancy Regulating a Complex Interplay between HMGA2/HIF1A and the Oncogenic Long Non-Coding RNA (lncRNA) NEAT1

**DOI:** 10.3390/ijms21103610

**Published:** 2020-05-20

**Authors:** Valentina Lulli, Mariachiara Buccarelli, Ramona Ilari, Giorgia Castellani, Chiara De Dominicis, Alessandra Di Giamberardino, Quintino Giorgio D′Alessandris, Stefano Giannetti, Maurizio Martini, Vittorio Stumpo, Alessandra Boe, Gabriele De Luca, Mauro Biffoni, Giovanna Marziali, Roberto Pallini, Lucia Ricci-Vitiani

**Affiliations:** 1 Department of Oncology and Molecular Medicine, Istituto Superiore di Sanità, 00161 Rome, Italy; valentina.lulli@iss.it (V.L.); mariachiara.buccarelli@iss.it (M.B.); ramona.ilari@iss.it (R.I.); giorgia.cast@hotmail.it (G.C.); chiara.dedominicisjob@gmail.com (C.D.D.); aledigiamberardino@libero.it (A.D.G.); gabriele.deluca@iss.it (G.D.L.); mauro.biffoni@iss.it (M.B.); 2 Department of Neuroscience, Institute of Neurosurgery, Università Cattolica del Sacro Cuore, Fondazione Policlinico Universitario A. Gemelli IRCCS; 00168 Rome, Italy; giorgiodal@hotmail.it (Q.G.D.); vittorio.stumpo@yahoo.it (V.S.); roberto.pallini@unicatt.it (R.P.); 3 Department of Neuroscience, Institute of Anatomy, Università Cattolica del Sacro Cuore, Fondazione Policlinico Universitario A. Gemelli IRCCS, 00168 Rome, Italy; stefano.giannetti@unicatt.it; 4 Department of Health Science and Public Health, Institute of Pathology, Università Cattolica del Sacro Cuore, Fondazione Policlinico Universitario A. Gemelli IRCCS, 00168 Rome, Italy; maurizio.martini@unicatt.it; 5 Core Facilities, Istituto Superiore di Sanità, 00161 Rome, Italy; alessandra.boe@iss.it

**Keywords:** MiR-370-3p, lncRNA NEAT1, HMGA2, HIF1A, glioblastoma, glioblastoma stem-like cells

## Abstract

Glioblastoma (GBM) is the most aggressive and prevalent form of a human brain tumor in adults. Several data have demonstrated the implication of microRNAs (miRNAs) in tumorigenicity of GBM stem-like cells (GSCs). The regulatory functions of miRNAs in GSCs have emerged as potential therapeutic candidates for glioma treatment. The current study aimed at investigating the function of miR-370-3p in glioma progression, as aberrant expression of miR-370-3p, is involved in various human cancers, including glioma. Analyzing our collection of GBM samples and patient-derived GSC lines, we found the expression of miR-370-3p significantly downregulated compared to normal brain tissues and normal neural stem cells. Restoration of miR-370-3p expression in GSCs significantly decreased proliferation, migration, and clonogenic abilities of GSCs, in vitro, and tumor growth in vivo. Gene expression analysis performed on miR-370-3p transduced GSCs, identified several transcripts involved in Epithelial to Mesenchymal Transition (EMT), and Hypoxia signaling pathways. Among the genes downregulated by the restored expression of miR-370-3p, we found the EMT-inducer high-mobility group AT-hook 2 (HMGA2), the master transcriptional regulator of the adaptive response to hypoxia, Hypoxia-inducible factor (HIF)1A, and the long non-coding RNAs (lncRNAs) Nuclear Enriched Abundant Transcript (NEAT)1. NEAT1 acts as an oncogene in a series of human cancers including gliomas, where it is regulated by the Epidermal Growth Factor Receptor (EGFR) pathways, and contributes to tumor growth and invasion. Noteworthy, the expression levels of miR-370-3p and NEAT1 were inversely related in both GBM tumor specimens and GSCs, and a dual-luciferase reporter assay proved the direct binding between miR-370-3p and the lncRNAs NEAT1. Our results identify a critical role of miR-370-3p in the regulation of GBM development, indicating that miR-370-3p acts as a tumor-suppressor factor inhibiting glioma cell growth, migration and invasion by targeting the lncRNAs NEAT1, HMGA2, and HIF1A, thus, providing a potential candidate for GBM patient treatment.

## 1. Introduction

Glioblastoma (GBM) is the most malignant primary brain tumor in adults with overall survival of 14-15 months [1,2]. Gold standard treatment for this tumor includes surgical resection and adjuvant therapy [2]. Although relatively rare in occurrence, this tumor has a relevant social impact because the peak incidence coincides with the age of professional maturity [1]. To improve therapeutic options, studies to identify and validate single protein targets are underway and, a number of novel treatments have been proposed based on therapeutic efficacy in vitro and in vivo. However, in most cases, targeted compounds that perform well in preclinical studies have failed for several major factors, including poor pharmacokinetic properties, suboptimal clinical trial design, emergence of resistance pathways, and inter- and intra-tumoral heterogeneity [3].

GBM is heterogeneous at different levels, including appearance, genetic composition and the variety of mechanisms by which it evades therapy [4]. Extensive studies of GBM have borne out the concept that within tumors, a small population of therapy-resistant and slow-dividing malignant cells exists, named GBM stem-like cells (GSCs). Therefore, GSCs have been directly linked as a major cause of therapeutic relapse and resistance to therapy [5].

Non-coding RNAs (ncRNAs) act as key regulators of physiological processes as well as, in pathological context. Particularly relevant in cancer, ncRNAs have been identified as oncogenic drivers and tumor suppressors in many cancers. NcRNAs can be divided into different classes, broadly based on their size. Small ncRNAs include microRNAs (miRNAs), transferRNA-derived small RNAs (tsRNAs) and PIWI-interacting RNAs (piRNAs). At the opposite end of the size are the long non-coding RNAs (lncRNAs), which are a very heterogeneous group of transcripts that have been identified as essential players in gene regulation by different mechanisms [6].

Currently, miRNAs and lncRNAs are the two most widely studied classes of ncRNAs. LncRNAs are RNAs with no functional protein-coding ability, associated with various biological processes such as epigenetic regulation, apoptosis, cell cycle, regulation of transcription and translation, cell growth, differentiation, and stem cell biology [7]. Thus far, lncRNAs are known to regulate gene expression at the transcriptional, post-transcriptional, and epigenetic levels. Some of them can serve as scaffolds to regulate protein-protein interaction, decoys to bind miRNAs, or guides to recruit epigenetic regulators on chromatin. LncRNAs, such as other ncRNAs, are deemed to influence global gene expression thus affecting a wide range of cellular processes due to the interactions with different molecules.

The dysregulated expression of lncRNAs is thought to increase the propensities of tumor development. They are found to be differentially expressed in tumors, where lncRNA dysregulation is associated with the cancer cell potential to initiate tumor growth, metastasis, and decreased patient survival in a wide range of cancers [8,9,10]. Studies so far suggest that many functional lncRNAs have the potential to act as oncogenes and/or tumor suppressors [9,11]. Recently, accumulating evidences have indicated that aberrant expression of lncRNAs may affect glioma initiation and progression [12,13]. Therefore, lncRNAs may act as biomarkers for glioma diagnosis, prognosis, and target therapy [14].

MiRNAs act at the post-transcriptional level by blocking mRNA translation and/or mRNA stability and by cleavage mRNAs and lncRNAs. Global analyses have revealed that several miRNAs are clinically implicated in GBM [15], with some reports indicating the association of miRNA dysregulation with acquired resistance to temozolomide (TMZ), the drug used for GBM standard chemotherapy [16,17,18]. Recent studies have shown that miRNAs could play a role in determining cancer stem cell properties, contribute to treatment resistance, and suggest that miRNAs are not only putative biological markers for diagnosis, but also promising targets for GBM treatment [19].

MiR-370-3p is located within the DLK1-DIO3 domain, which has been identified as a cancer-associated genomic region on human chromosome 14 [20]. Several studies reported that downregulation of miR-370-3p is involved in tumorigenicity in various types of cancers such as oral squamous carcinoma [21], hepatocellular carcinoma [22], acute myeloid leukemia [23], and ovarian cancer [24]. Other studies found that upregulation of miR-370-3p contributes to the progression of prostate cancer [25], gastric carcinoma [26], Wilms tumor [27]. For human malignant glioma, recent studies provided evidence that miR-370-3p is involved in GBM tumorigenesis [28,29]. MiR-370-3p expression was frequently found decreased in glioma tissues, particularly in recurrent GBM [28]. Moreover, it has been shown that this miRNA may act as tumor suppressor in glioma by inhibiting cell proliferation [29]. However, the biological role and functional mechanisms of this miRNA are still largely unknown.

In this study, we showed a critical role for miR-370-3p in the regulation of GBM development, suggesting that this miRNA might act as a tumor-suppressor factor. Targets of miR-370-3p are the Epithelial to Mesenchymal Transition (EMT)-inducer high-mobility group AT-hook 2 (HMGA2), the master transcriptional regulator of the adaptive response to hypoxia, Hypoxia-inducible factor (HIF)1A, and the lncRNAs Nuclear Enriched Abundant Transcript (NEAT)1. NEAT1 is critical for glioma cell growth and invasion, thus suggesting novel therapeutic interventions. Our study provides evidence of a potential therapeutic role of miR-370-3p in the treatment of GBM.

## 2. Results

### 2.1. MiR-370-3p is Downregulated in GBM and Its Restoration Impairs Tumorigenic Ability of GSCs

**Figure 1:**
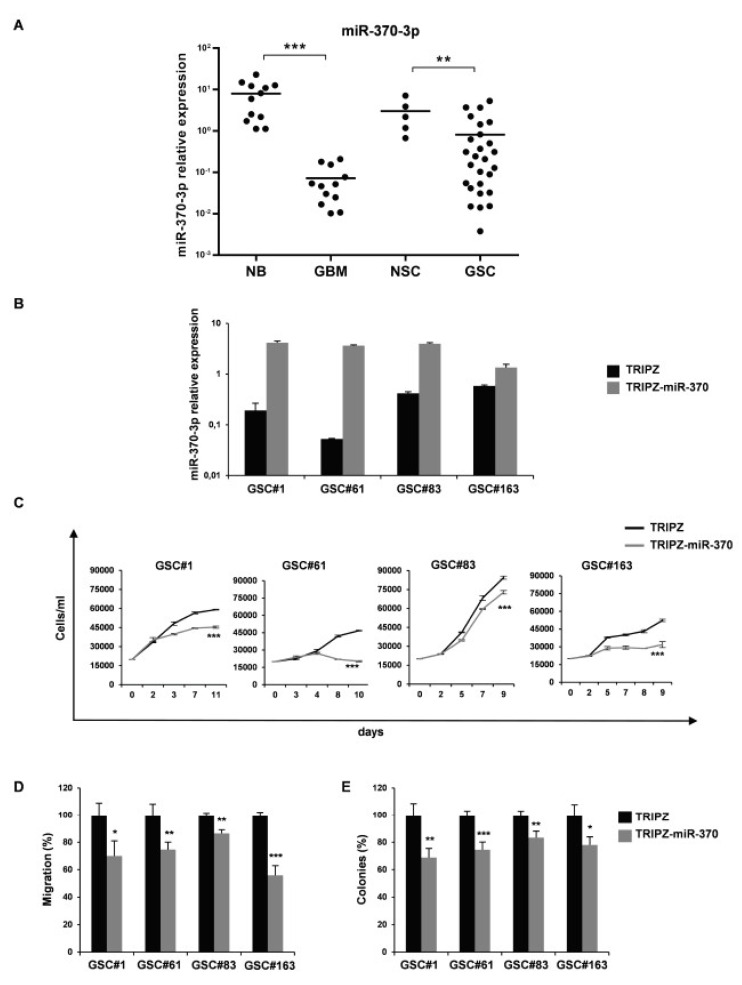
miR-370-3p is down-regulated in Glioblastoma (GBM) tissues and Glioblastoma Stem-like Cell (GSC) lines and its restoration reduces cell growth, migration and clonogenic abilities of GSCs. (**A**) Real time RT-PCR analysis of miR-370-3p expression performed on normal brain tissue samples (NB, *n* = 12), GBM tissues (GBM, *n* = 12), NSCs (*n* = 5) and GSCs (*n* = 27). Samples were run in duplicate and normalized with Glyceraldehyde-3-Phosphate Dehydrodenase (GAPDH). * *p* < 0.05; ** *p* < 0.01; *** *p* < 0.001. (**B**) Real time PCR analysis of miR-370-3p expression in GSC#1, #61, #83 and #163 transduced with either TRIPZ or TRIPZ-miR-370 inducible vectors. (**C**) Growth curves of GSCs transduced with either TRIPZ or TRIPZ-miR-370 vector. Points and range lines at each day represent mean and SD of at least two independent experiments in triplicate. Two-way analysis of variance for repeated measures was performed on the whole set of data. (**D**) Analysis of migration efficiency in GSCs transduced with TRIPZ-miR-370 vector 48 h after induction. Values are reported as percentage relative to control vector and shown as mean ± SD from two independent experiments in triplicate. (E) Analysis of efficiency in colony formation of GSCs after transduction with TRIPZ-miR-370 vector. Percent colony number values from two independent experiments in triplicate were calculated over the correspondent empty vector and are shown as mean ± SD for each GSC line.

In order to clarify the role of miR-370-3p in GBM, we analyzed its expression in 12 human GBM tissues and 27 GSCs derived from GBM surgical specimens. Clinical and pathological features are summarized in Table S1. As shown in Figure 1A, the expression level of miR-370-3p was significantly down-regulated in GBM tissues (*p* < 0.0006, Student’s t-test) and GSCs (*p* < 0.0083, Student’s t-test) compared to normal brain tissues (*n* = 12) and normal neural stem cells (NSCs, *n* = 5), respectively.

To further investigate the role of miR-370-3p on GSC tumorigenic properties, we restored miR-370-3p expression in GSC lines by using an inducible Tet-On lentiviral vector (TRIPZ) carrying pri-miR-370 in the 3′-untranslated region of Red Fluorescent Protein (RFP). Four GSC lines (#1, #61, #83, and #163) chosen as representatives of different miR-370-3p expression levels, were transduced and exposed to doxycycline. RFP-positive cells were flow-sorted and miR-370-3p restoration was confirmed by real-time PCR (Figure 1B). All the GSC lines transduced with this vector showed miR-370-3p expression levels comparable to normal cells or increased when compared to control vector (TRIPZ)-transduced cells.

Ectopic expression of miR-370-3p significantly impaired cell viability of all the GSC lines tested (Figure 1C).

We then examined whether miR-370-3p could alter additional malignant features of GSCs, such as migration. After miR-370-3p induction, the motility of transduced GSCs was examined and a dramatic reduction in the migration capabilities of TRIPZ-miR-370 GSCs (Figure 1D), was observed.

Moreover, we analyzed the clonogenic capability of GSC expressing miR-370-3p. Doxycycline-induced cells were plated as single cells in 96-well plates in triplicate and allowed to grow for two weeks. TRIPZ-miR-370 GSCs formed significantly fewer colonies as compared to TRIPZ cells (Figure 1E). Thus, miR-370-3p restoration resulted in a considerable inhibition of cell viability, migration and colony formation of all the GSCs tested, suggesting that this miRNA could play a pivotal role in GBM oncosuppression.

### 2.2. Restoration of miR-370-3p Inhibits Tumor Growth in Orthotopic Xenograft Mouse Models

**Figure 2:**
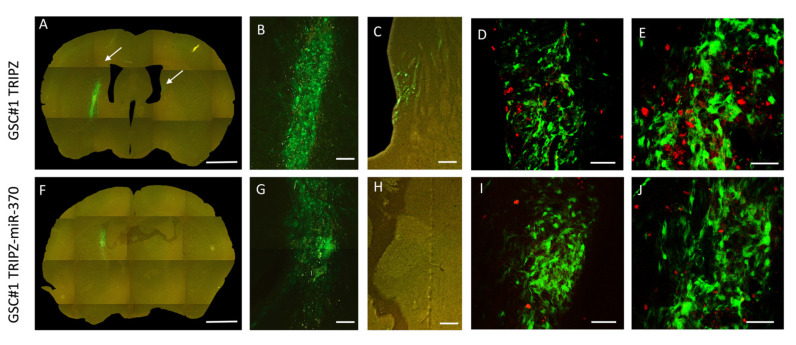
Restoration of miR-370 inhibits tumor growth in orthotopic xenograft mouse models. Fluorescence microscopy of mouse brain grafted onto the right striatum with GSC#1 cells. *Upper panel*, Photomontage of coronal section in a TRIPZ xenograft (**A**) showing the injection area (**B**) and tumor spreading to the subventricular zone of the contralateral hemisphere (**C**). The arrows point to tumor cells. *Lower panel*, Photomontage of coronal section in a TRIPZ-miR-370 xenograft (**F**). A few tumor cells with cell debris are found in the injection area (**G**). There is no contralateral spreading of tumor cells (**H**). Scale bars. A and F, 1 mm; B and G, 135 μm; C and H, 150 μm. Ki67 immunostaining showing positive nuclei of proliferating cells (*red*) in a TRIPZ xenograft (**D**–**E**) and in a TRIPZ-miR-370 xenograft (**I**–**J**). Scale bars. D and I, 90μm; E and J, 70 μm.

Following orthotopic injection into immunocompromised mice, patient-derived GSCs generate highly infiltrative tumors that closely mimic the parent neoplasm [30]. Therefore, we utilized this model to test in vivo the effects of miR-370-3p restoration on GBM growth. To trace the grafted cells in vivo we transduced both TRIPZ and TRIPZ-miR-370 GSC#1 with a green fluorescence protein (GFP) expressing lentiviral vector. At 4 weeks after grafting, control mice (*n*, 3) grafted with TRIPZ GSC#1 cells harbored tumors that invaded the injection site in the striatum and homolateral piriform cortex (Figure 2, and Figure S1). Some tumor cells spread through the corpus callosum to the contralateral paraventricular area (Figure 2A-C and Supplementary Figure S1). In mice grafted with TRIPZ-miR-370 GSC#1 cells (*n*, 3), the degree of brain invasion of the injected striatum was reduced (Figure 2F-H and Supplementary Figure S1). We did not observe tumor cells spreading to the contralateral hemisphere. The volume of the brain invaded by tumor cells was 133.54 × 10^6^ ± 20.50 µm^3^ (mean ± sem) and 34.89 × 10^6^ ± 8.49 µm^3^ in mice grafted with TRIPZ GSC#1 cells and in those grafted with TRIPZ-miR-370 GSC#1 cells, respectively (*p* = 0.027, Student-*t* test).

To assess the impact of miR-370 on GSCs proliferation, we used the Ki67 labeling index. Consistently, proliferation of tumor cells was 23.78 ± 1.99 percent and 10.56 ± 0.88 percent in control TRIPZ GSC#1 brain xenografts and in TRIPZ-miR-370 GSC#1 grafts, respectively (*p* < 0.0001; Student-*t* test; Figure 2D,E,I,J).

### 2.3. Tumor Suppressor Function of miR-370-3p Involved Inhibition of EMT and Response to Hypoxia in GSCs

To further investigate the miR-370-3p regulatory network and to identify main mediators of its tumor suppression function, we analyzed gene expression profiles of GSC#1 cells transduced with TRIPZ-miR-370 or TRIPZ empty vector by microarray. We hence examined the annotation for the most deregulated genes. To detect significant variations in gene networks, we explored the Molecular Signature DataBase (MSigDB), a collection of annotated gene sets, with Gene Set Enrichment Analysis (GSEA) software (https://www.gsea-msigdb.org/gsea/index.jsp) [31,32].

**Figure 3:**
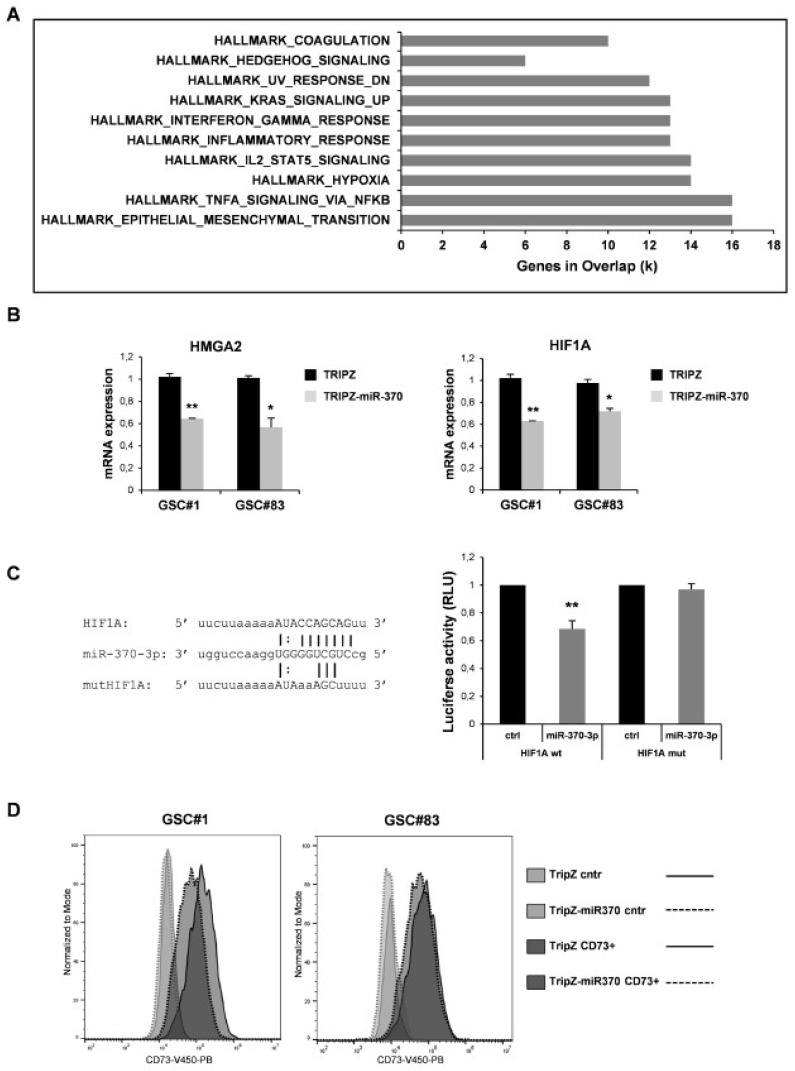
Effect of miR-370 restoration in GSCs. (**A**) Pathway enrichment analysis examined by GSEA software of highly modulated genes (≥2 fold both up and down) in GSC#1 transduced with TRIPZ-miR-370 vs TRIPZ empty vector. (**B**) RT-PCR analysis performed on GSC#1 and GSC#83 transduced with TRIPZ or TRIPZ-miR-370 for HMGA2 (left panel) and Hypoxia-inducible factor (HIF) 1A expression (right panel). * *p* < 0.05; ** *p* < 0.01. (**C**) Predicted binding site of miR-370-3p in the HIF1A 3′UTR sequence (left panel). Dual-luciferase reporter assay of pGL3 vectors containing the HIF1A 3′UTR or HIF1A mutant (mut), co-transfected in 293T cells with hsa-miR-370-3p-mimic or control mimic RNA (ctrl). ** *p* < 0.01. Histograms show normalized mean values of the relative luciferase activity. Error bars represent the mean ± SD (*n* = 4) (right panel) (**D**) FACS analysis of NT5E/CD73 on GSC#1 and GSC#83 transduced with TRIPZ empty vector and TRIPZ-miR-370 after doxycycline induction.

We searched among the transcripts that resulted deregulated, at least to two-fold (both up and down ≥ 2 folds), in the miR-370-3p transduced cells for enrichment in specific pathways. We analyzed 836 unique identifiers corresponding to the down- or up-regulated transcripts with the functional annotation-clustering tool. Eighty-one genes were identified, assigned to pathways enriched with a *p*-value below 0.001. The most significantly enriched pathways were related to EMT and hypoxia (Figure 3A and Table S2).

A list of the potential target mRNAs for miR-370-3p was obtained using TargetScan 6.2 and microRNA.org algorithms (www.targetscan.org, http://www.microrna.org/microrna/home.do). Among these, the high-mobility group AT-hook 2 (HMGA2) belongs to the high-mobility group (HMG) protein family and is reported to function as an oncogene and EMT inducer in several types of human cancer including glioma, where its over-expression was revealed to be closely associated with aggressive cell behaviors [33]. The targeting of HMGA2 by miR-370-3p was already described in the context of no-functional pituitary adenoma [34]. MiR-370-3p restoration induced a significant decrease of HMGA2 expression in both GSC lines analyzed indicating a direct targeting also in the GBM context (Figure 3B, left panel). Among the other potential miR-370-3p targets we noticed Hypoxia-inducible factor (HIF) 1A. Restored expression of miR-370-3p in GSCs significantly reduced the level of HIF1A mRNA in the TRIPZ-miR-370 compared to TRIPZ empty vector cells (Figure 3B, right panel).

To prove the direct targeting of HIF1A by miR-370-3p, a dual-luciferase reporter assay was performed. Wild type (wt) HIF1A and mutant (mut) HIF1A vectors were constructed (Figure 3C, left panel) for the assay and we found that miR-370-3p significantly reduced the luciferase activity of wt HIF1A but not of the mut HIF1A reporter (Figure 3C, right panel).

Interestingly, among the most significantly down-regulated genes in miR-370 restored GSCs we found NT5E/CD73, an enzyme responsible for adenosine (ADO) production. It has recently been shown that CD73 down-regulation decreased glioma cell migration and invasion and CD73 knockdown potentiated TMZ cytotoxic effect on glioma cells by decreasing the IC50 value and sensitizing cells to a non-cytotoxic drug concentration [35]. Although CD73 was not found to be a direct target of miR-370-3p, its expression was reduced in TRIPZ-miR-370 compared to TRIPZ empty vector cells as assessed by flow cytometry (Figure 3D). These results suggest that miR-370-3p may indirectly modulate CD73 expression by directly regulating HIF1A expression as HIF1A binds to the NT5E promoter thereby activating CD73 expression [36,37].

### 2.4. MiR-370-3p Interacts with NEAT1 and Their Expression is Inversely Correlated in GBM Tissues and GSC Lines

**Figure 4:**
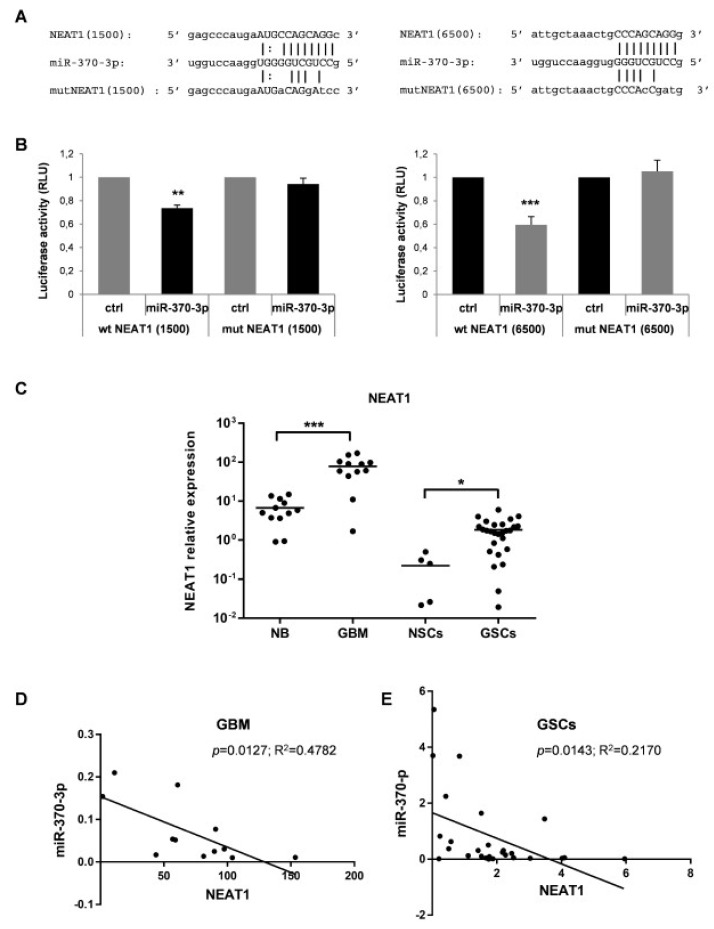
Nuclear Enriched Abundant Transcript 1 (NEAT1) is a target of miR-370-3p and its expression in GBM tissues and GSC lines is inversely correlated with miR-370-3p. (**A**) Binding sites between miR-370-3p and NEAT1 at +1500 (left panel) and at +6500 (right panel) nucleotides of RNA sequence. (**B**) Dual-luciferase reporter assays in 293T cells co-transfected with reporter vectors containing the wild type (wt) or the mutated (mut) NEAT1(1500) (left panel) or NEAT1(6500) (right panel) sequences and miR-370-3p-mimic or control mimic RNA (ctrl). Bar charts show normalized mean values of the relative luciferase activity. Error bars represent the mean ± SD (*n* = 4) (student t-test: ** *p* < 0.01; *** *p* < 0.001. (**C**) RT-PCR analysis performed on normal brain samples (NB, *n* = 12), GBM tissues (GBM, *n* = 12), NSCs (*n* = 5) and GSCs (*n* = 27). Samples were run in duplicate and normalized with GAPDH. * *p* < 0.05; ** *p* < 0.01; *** *p* < 0.001. (**D**) Correlation analysis between miR-370-3p and NEAT1 expression levels in GBM tissues and (**E**) in GSC lines.

Growing evidences have confirmed that miRNAs and lncRNAs can interact with each other in a sequence-specific manner [38]. To identify lncRNAs with complementary base pairing with miR-370-3p, we used the online software starBase v2.0 (http://starbase.sysu.edu.cn) [39]. Among the predicted lncRNAs that may interact with miR-370-3p, we focused our attention on lncRNAs Nuclear Enriched Abundant Transcript 1 (NEAT1) for further investigation since NEAT1 is linked to hypoxia [40,41,42], promotes EMT [42] and correlates with higher World Health Organization (WHO) grade human glioma tissues [43]. Dual-luciferase reporter assays were performed to prove the direct binding between NEAT1 and miR-370-3p in the two predicted seeds (chr11:65191743-65191764[+] and chr11:65196739-65196760[+]) at +1500 and +6500 nucleotides of the RNA sequence, respectively. (Figure 4A). The wild type (wt) NEAT1 and mutant (mut) NEAT1 reporter vectors were co-transfected in 293T cells with miR-370-3p mimics or with non-targeting control RNA (ctrl) (Figure 4A). The miR-370-3p mimic significantly reduced the luciferase activity of wt NEAT1 but not of the mut NEAT1 reporter constructs both for NEAT1( + 1500) and for NEAT1(+6500) (Figure 4B).

Therefore, we examined the NEAT1 expression levels in the same cohort of samples analyzed for miR-370-3p expression i.e., 12 human GBM tissues and in 27 GSC lines compared to 12 normal brain tissues and 5 normal NSCs. As shown in Figure 4C, the expression level of NEAT1 was significantly elevated in GBM tissues (*p* < 0.0001, Student’s t-test) and GSCs (*p* < 0.017, Student’s t-test) compared to normal brain tissues and normal NSCs, respectively.

Noteworthy, miR-370-3p and NEAT1 expression levels are inversely correlated in both GBMs (Figure 4D) and GSCs (Figure 4E) (Spearman correlation coefficient 0.63 and 0.22, *p* < 0.006 and *p* < 0.01, respectively) confirming that there may be a reciprocal regulation between miR-370-3p and NEAT1.

Unlike other types of tumor, i.e., urothelial bladder cancer (r = −0.26), head and neck squamous cell carcinoma (r = −0.29), acute myeloid leukemia (r = −0.18), we did not found significant correlation between these ncRNAs in GBM patients present in the The Cancer Genome Atlas (TCGA) dataset (http://starbase.sysu.edu.cn/starbase2/).

**Figure 5:**
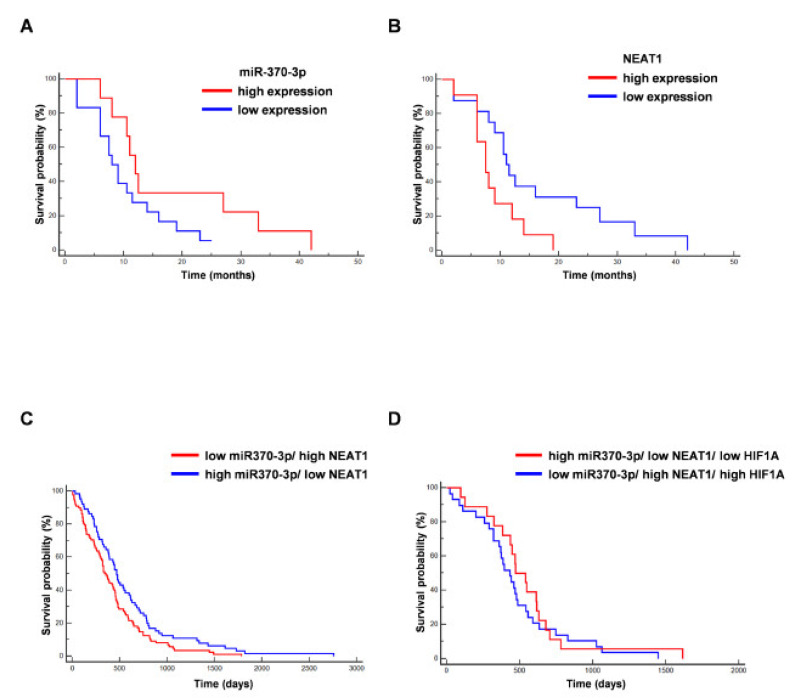
Correlation between miR-370-3p and NEAT1 expression and overall survival (OS) of GBM patients. (**A**) Kaplan–Meier curve for OS in our cohort of GBM patients stratified for low miR-370-3p expression (<the median value) *vs* high miR-370-3p expression (>the median value). The last group was not associated to a better outcome (*n* = 27, *p* = 0.09, Hazard Ratio (HR) 0.4778, 95% CI from 0.2029 to 1.1253). (**B**) Kaplan–Meier curve for OS in our cohort of GBM patients stratified for low NEAT1 expression (<the median value) vs. high NEAT1 expression (>the median value). The last group was significantly associated to a worse outcome (*n* = 27, *p* = 0.035, HR 2.7980, 95% CI from 1.0754 to 7.2804). (**C**) Kaplan–Meier curve for OS in GBM The Cancer Genome Atlas (TCGA) patients stratified for low miR-370-3p/ high NEAT1 vs. high miR-370-3p/ low NEAT1. The last group was significantly associated to a better outcome (*n* = 153, *p* = 0.0215, HR 0.6855, 95% CI from 0.4968 to 0.9458). (**D**) Kaplan–Meier curve for OS in GBM TCGA patients stratified for low miR-370-3p/high NEAT1/high HIF1A *vs* high miR-370-3p/low NEAT1/low HIF1A (*n* = 47, *p* = 0.4400, HR 0.7927, 95% CI from 0.4395 to 1.4296).

In line with previously published results, we found that the expression level of miR-370-3p was not associated with prolonged value for survival in the analyzed GBM patients (from which GSCs are derived) (*p* = 0.0911; Hazard Ratio (HR) 0.4778; 95% CI from 0.2029 to 1.1253) [44], while high NEAT1 expression was associated to unfavorable overall survival (OS) (*p* = 0.035; HR 2.7980; 95% CI from 1.0754 to 7.2804) [43] (Figure 5A,B).

Further, we analyzed the association of miR-370-3p and NEAT1 expression with survival of TCGA database patients. Figure 5C shows the Kaplan–Meier curve for OS in GBM TCGA patients stratified for low miR-370-3p/high NEAT1 vs. high miR-370-3p/low NEAT1. The last group was significantly associated to a better outcome (*p* = 0.0215, HR 0.6855, 95% CI from 0.4968 to 0.9458). Conversely, including the analysis, the expression levels of HIF1A (Figure 5D), the Kaplan–Meier curve for OS in GBM TCGA patients stratified for low miR370-3p/ high NEAT1/ high HIF1A vs. high miR370-3p/ low NEAT1/low HIF1A (*p* = 0.4400, HR 0.7927, 95% CI from 0.4395 to 1.4296) shows that the last subgroup is not significantly correlated to a better prognosis, although there is an association with patients with longer survival.

**Figure 6:**
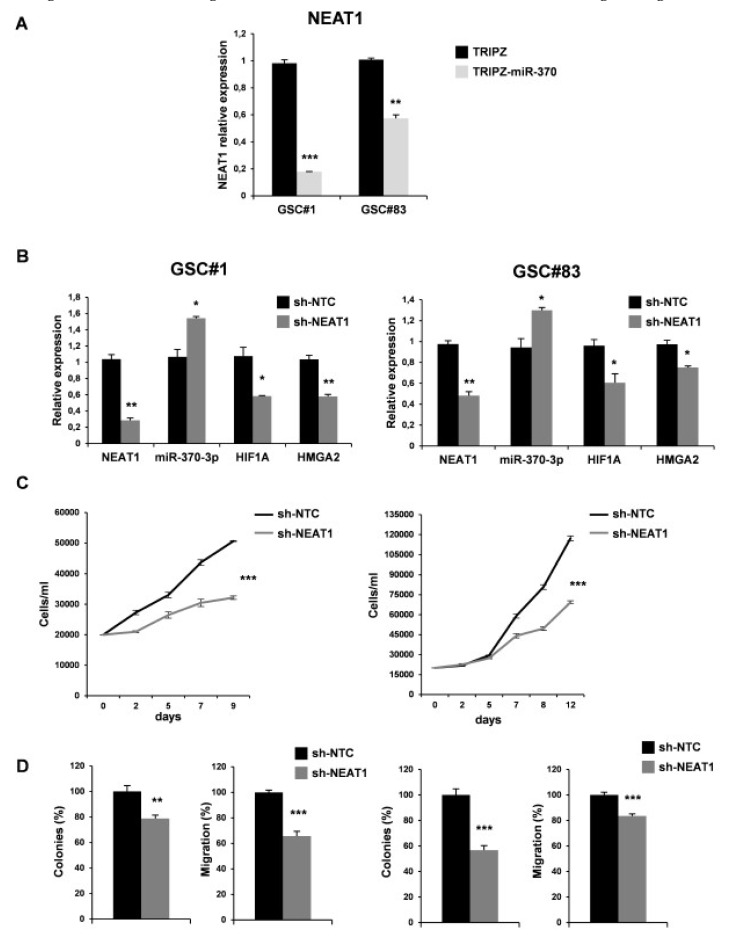
NEAT1 knockdown impairs GSC tumorigenicity. (**A**) Real time PCR analysis for NEAT1 on GSC#1 and GSC#83 miR-370-3p transduced lines confirmed the down-regulation of the lncRNA. ** *p* < 0.01; *** *p* < 0.001. (**B**) Real time PCR for NEAT1, miR-370-3p, HIF1A and HMGA2 on GSC#1 (left panel) and GSC#83 (right panel) transduced with short hairpin (sh)NEAT1 or its no target control lentiviral vector (shNTC). * *p* < 0.05; ** *p* < 0.01. (**C**) Growth curves, (**D**) analysis of efficiency in colony formation and analysis of migration efficiency in GSC#1 (left panel) and GSC#83 (right panel) transduced with shNEAT1 or shNTC. Values shown are mean ± SD from two independent experiments in triplicate. ** *p* < 0.01; *** *p* < 0.001.

As expected, miR-370-3p restoration induced a significant down-regulation of NEAT1 confirming a direct interaction between the two non-coding RNAs (Figure 6A).

We then examined the effect of NEAT1 silencing by transducing lentiviral vector carrying short hairpin (sh)NEAT1 or shNTC (no target control) sequence and green fluorescent protein (GFP) as a reporter in GSC#1 and GSC#83. After transduction, a 40% decrease of the endogenous levels of NEAT1 was observed in GFP-positive cells (Figure 6B). We then evaluated the effect on viability, clonogenic ability, and migration in vitro (Figure 6C-D). As expected, NEAT1 knockdown induced a significant decrease of cell viability (Figure 6C). To analyze the clonogenic ability, shNEAT1, and shNTC transduced GSCs were plated and grown as single cells. The clonogenic ability of stably NEAT1 silenced GSCs was reduced in all the lines compared to the shNTC GSCs (Figure 6D). Furthermore, a significant decrease in the migration of the NEAT1-silenced cells compared to control was found (Figure 6D), confirming the role of this lncRNA as a critical effector of gliomagenesis.

**Figure 7:**
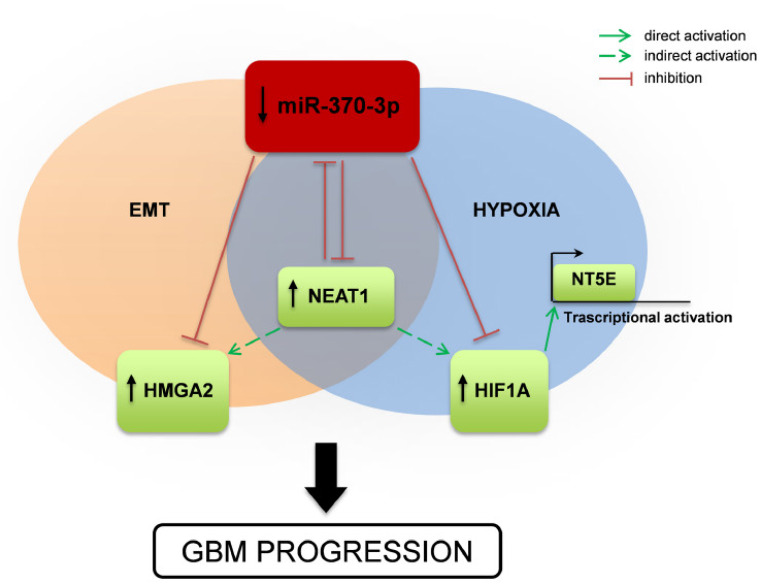
Schematic drawing of miR-370-3p regulation of HMGA2-HIF1A-NEAT1 interplay and effect on GBM tumorigenesis. Representation of the mechanism proposed in the present study. miR-370-3p down-regulation leads to increased expression of the lncRNA NEAT1, the Epithelial to Mesenchymal Transition (EMT)-inducer HMGA2, the master transcriptional regulator HIF1A, which in turn activates NT5E expression. NEAT1 inhibits the expression of miR-370-3p, promoting the up-regulation of HMGA2 and HIF1A and contributing to GBM progression.

Altogether our results suggest a complex interplay among different species of RNAs (Figure 7), in which miR-370-3p displays a tumor-suppressor function in GSCs by targeting mRNAs involved in EMT and in hypoxia (i.e., HMGA2 and HIF1A, respectively). HIF1A in turn regulates transcription of NT5E/CD73 whose involvement in tumor development is widely documented as well as those in adaption to hypoxia and promotion of immune-escape. Moreover, miR-370-3p exerts its function by targeting the lncRNA NEAT1 that is critical for GBM cell growth and invasiveness. Interestingly, hypoxic induction of NEAT1 promotes cell proliferation and survival and inhibits apoptosis [41].

## 3. Discussion

MiR-370-3p has an important role as a tumor suppressor gene that is associated with the genesis and progression of human gliomas. Previous studies shows that miR-370-3p is down-regulated in human glioma cells [29] and its over-expression restrained GBM progression, including cell proliferation, migration and invasion, while promoting apoptosis [45]. MiR-370-3p inhibited the proliferation of human glioma cells by regulating the levels of β-catenin and the activation of FOXO3a [29,46]. A recent report showed that up-regulation of miR-370-3p sensitizes GBM cells to TMZ [28].

Our findings add an additional role for miR-370-3p in brain tumors, in that restoration of miR-370-3p expression inhibited cell growth, migration, and colony-forming ability of GSCs in vitro and decreased the growth of GSC-derived tumors in vivo. Moreover, we explored the complex interplay between different species of RNAs, in which miR-370-3p exerts a tumor-suppressor function in GSCs by targeting HMGA2 and HIF1A mRNAs, genes involved in EMT and hypoxia.

HMGA2 is a member of the non-histone chromosomal high mobility group (HMG) protein family, it mainly works as transcriptional regulating factor by altering chromatin architecture [47] playing an important role in EMT in several tumors [48,49].

One hallmark of GBM is the intense cell proliferation and inadequate vascularization that causes the presence of hypoxic regions around the necrotic core of the tumor with insufficient oxygen supply [50]. Hypoxia drives the sustenance and expansion of GSCs in the tumor [50]. Hypoxia also enhances the invasiveness of tumor cells via EMT, which exacerbates GBM aggressiveness, by inducing a mesenchymal shift that is mediated by the HIF1A-ZEB1 axis leading to an elevated invasive potential [51].

Noteworthy, among the most down-regulated genes in miR-370-3p restored GSCs we found NT5E/CD73, a protein bound to the outer surface of the plasma membrane by a glycosylphosphatidylinositol (GPI) anchor and localized within lipid rafts [52]. CD73 is an ecto-5′-nucleotidase hydrolyzing extracellular adenosine monophosphate (AMP) into adenosine and inorganic phosphate [53], whose over-expression has been reported in a variety of cancer cells and tumor patient biopsies, including gliomas, being associated with worse disease-free survival in GBM patients [54].

CD73 plays a role in the control of glioma cell migration and invasion through an adenosinergic pathway. CD73 downregulation decreased glioma cell migration and invasion by reducing metalloproteinase-2 and vimentin expression and reduced cell proliferation. Those effects also involved AKT/NF-kB pathway. Moreover, CD73 knockdown or enzyme inhibition potentiated TMZ cytotoxic effect on glioma [35].

Interestingly, HIF1A can directly bind to the NT5E promoter, activating its expression [36], confirming a role for NT5E in hypoxia adaptation [37] even in GBM. Thus, hypoxia resulting from uncontrolled tumor cell proliferation might induce HIF1A mediated up-regulation of NT5E expression on GSCs. Moreover, miR-370-3p exerts its function by targeting the lncRNA NEAT1. LncRNA NEAT1 is found to act as an oncogene in the majority of human cancers [55,56,57,58,59,60], including GBM [61].

In terms of carcinogenesis, NEAT1 mainly functions as competing endogenous RNA (ceRNA) by sponging tumor-suppressive miRNAs [62]. Subsequently, these miRNAs lose the ability to function as a tumor suppressor and oncogenic mRNAs are translated contributing to tumorigenesis [63]. Moreover, as several lncRNAs, which exert their functions by binding to both promoter and distal regulatory elements, NEAT1 controls 13.3% of genes in the PI3K-AKT signaling pathway by interacting with distal regulatory elements [64].

In GBM, NEAT1 over-expression correlated with clinic-pathological characteristics, such as larger tumor size, higher WHO grade, recurrence, and with a poor prognosis in glioma patients [43]. In line with these clinical results, over-expression of NEAT1 conferred malignancies to glioma, and NEAT1 knockdown inhibited glioma cell proliferation, invasion, and migration [65]. Mechanistic study showed that NEAT1 serves as a competitive endogenous lncRNA sponging miR-449b-5p, inducing c-Met expression thus potentially contributing to the development of glioma [65].

It was found that in GBM NEAT1 levels were regulated by Epidermal Growth Factor Receptor EGFR pathway activity, which was mediated by STAT3 and NF-κB (p65) downstream of the EGFR pathway. Moreover, NEAT1 was critical for glioma cell growth and invasion by increasing β-catenin nuclear transport and down-regulating ICAT, GSK3B, and Axin2 [61].

Our data confirm the oncogenic role of NEAT1 and the tumor suppressor role of miR-370-3p in GBM by using patient-derived GSCs, which represent a valuable tool to obtain data to be translated onto clinical settings. The translational value of patient-derived GSCs was recently described in study showing that GSCs predict the response of the parent tumor to current GBM therapies (i.e., radiation and TMZ) [30].

The lncRNA–miRNA–mRNA crosstalk represents a regulatory key for the maintenance of cellular homeostasis. In every major cancer type, disruption of non coding RNA (ncRNA) regulatory networks has already been described to have oncogenic or tumor-suppressive effects. Likewise, in GBM, the lncRNA-miRNA co-expression network contributes to GBM onset, progression, angiogenesis, and drug resistance. The complex interplay coordinated by miR370-3p in GSCs is schematically showed in Figure 7.

The evidences on critical role of miRNAs in the formation and progression of glioma enable some of them to emerge as clinical markers in diagnosis, prognosis, as well as novel agents in mitigation of GBM. Recently, advancements on oligonucleotides delivery (selective targeting with Aptamers, Cell-Penetrating Peptides, and Nanoparticles) to the brain and malignant gliomas via local and systemic have been achieved [66]. Particularly, different types of nanoparticles [67], have been rationalized for optimal benefit in different pathological states including neuronal cancers. These delivery systems not only protect miRNAs from degradation by nucleases but also increases their half-life in the blood or serum. Moreover, numerous clinical trials (http://clinicaltrials.gov) reported combination therapies as promising method for GBM treatment, and nanotechnology allowing conjugation of miRNAs with anti- cancer drugs enabled delivery of miRNAs across the Blood Brain Barrier (BBB)either alone or in combinations.

A deeper understanding of the complex networks of interactions that ncRNAs coordinate will lead to a greater understanding of the potential for the application of ncRNAs as targets of cancer therapy and diagnostic.

## 4. Methods

### 4.1. GBM and Normal Brain Tissue Sample Collection and Cell Cultures

All human GBM and normal brain tissues were collected from adult patients who underwent surgery for complete or partial resection of GBM or craniotomy for non-tumoral pathology, at the Institute of Neurosurgery, Catholic University School of Medicine in Rome. All the patients provided written informed consent according to the research proposals approved by the Ethical Committee of the Catholic University School of Medicine (no. 1720). A diagnosis of GBM was established histologically by the neuropathologist in accordance with the WHO classification [68].

Glioblastoma stem-like cells were isolated from surgical samples subjected to mechanical dissociation and the resulting cell suspension was cultured in a serum free medium supplemented with EGF and basic Fibroblast Growth Factor (bFGF) as previously described [69]. GSC lines were validated by Short Tandem Repeat (STR) DNA fingerprinting. Nine highly polymorphic STR loci plus amelogenin (Cell ID™ System, Promega Inc., Madison, WI) were used. Detection of amplified fragments was obtained by ABI PRISM 3100 Genetic Analyzer (Applied Biosystems, Carlsbad, CA, USA). Data analysis was performed by GeneMapper^®^ software, version 4.0 (Biological Bank and Cell Factory, National Institute for Cancer Research, IST, Genoa, Italy). All GSC profiles were challenged against public databases to confirm authenticity. Human adult neural stem cell line, was isolated from human neural adult tissue obtained following the ethical guidelines of the NECTAR and the Declaration of Helsinki from patients undergoing particularly invasive neurosurgery as previously described [70].

Human neural progenitor cell (HNPC) lines were purchased from Lonza (Lonza Inc., Walkersville, MD, USA).

Packaging cell line, 293T, was maintained in DMEM (Life Technologies Corporation, Carlsbad, CA, USA) supplemented with 10% (*v*/*v*) heat-inactivated FBS, 2 mM L-glutamine, 100 U/mL of penicillin and 100 µg/mL of streptomycin (Invitrogen, Carlsbad, CA, USA).

### 4.2. RNA Extraction and Quantitative Real-Time PCR (qRT-PCR)

Total RNA was extracted from cells using TRIzol reagent (Life Technologies Corporation) and reverse transcribed by Moloney Murine Leukemia Virus enzyme (M-MLV) with random primers (all from Invitrogen).

Real-time PCR for NEAT1, HMGA2, and HIF1A mRNA detection were performed with SYBR^™^ Green Master Mix in StepOnePlus™ Real-Time PCR System (Applied Biosystems™) and normalized with GAPDH using the primers listed in Table 1.

Analysis of mature miR-370-3p was performed using TaqMan^®^ miRNA Assay protocol (assay ID 002275, Applied Biosystems) and normalized with RNU6B (Assay ID: 001093, Applied Biosystems). All RT-PCR reactions were run in duplicate in StepOne Real-Time PCR System (Applied Biosystems, Foster City, CA, USA).

### 4.3. Flow Cytometry

For CD73 expression, TRIPZ and TRIPZ-miR-370 GSCs were incubated with the antibody for 30 min at RT, washed with PBS and analyzed by CytoFLEX LX flow cytometer (Beckman Coulter, Brea, CA, USA) equipped with a CytExpert software (Beckman Coulter Life Science, Milan, Italy). The antibody used was Brilliant Violet (BV) 421-conjugated mouse anti-human CD73/NT5E antibody or BV421-conjugated mouse IgG_1_ isotype control antibody (BD Biosciences, Milan, Italy). The staining was performed after doxycycline induction.

### 4.4. Plasmid Constructs and Lentivirus Infection

The miR-370-3p precursor was cloned in the 3′-untranslated (UTR) region of RFP in the TRIPZ doxycycline inducible lentiviral vector (ThermoFisher Scientific, Waltham, MA, USA). Primers used for pri-miRNA-370 amplification were: 5′-CTACTCGAGGGATGGGCGATAGTTCAGGT-3′ (Forward) and 5′-TATGCGGCCGCGCCCGAGCTCTGGTGTTA-3′ (Reverse). The short hairpin (sh)RNA sequence targeting NEAT1 (5′-gatccctaagctgtagaacat-3′, DOI: 10.1002/jcp.27093) was synthesized and cloned in GFP-C-shLenti-vector from OriGene (OriGene Technologies, Inc., Rockville, MD, USA).

Lentiviral particles were produced by the calcium phosphate transfection protocol in 293T packaging cell line and infection performed as previously described [70]. After infection for TRIPZ and TRIPZ-miR-370 vectors, transduced cells were selected with puromycin and Red Fluorescent Protein (RFP) fluorescence was evaluated by FACSCanto (BD Biosciences) upon doxycycline induction (Sigma Aldrich Inc., Saint Louis, MO). For shNTC and shNEAT vectors, Green Fluorescent Protein (GFP) fluorescence of transduced cells was evaluated by FACSCanto (BD Biosciences) and GFP-positive cells were flow sorted by FACS ARIA (BD Biosciences).

### 4.5. Cell Growth, Migration, and Colony Formation Assay

For the viability assay TRIPZ and TRIPZ-miR-370 GSCs, or shNTC-GFP and shNEAT1-GFP GSCs were plated at density of 2 × 10^4^/mL in 96-well plates in triplicate. Cell viability was monitored by counting the cells and confirmed by using the CellTiter-Blue Viability Assay (Promega).

The abilities of migration and invasion of transduced GSCs were evaluated by plating in Corning FluoroBlok™ Multiwell Inserts System (Corning Life Sciences, Tewksbury, MA, USA), according to manufacturer′s instruction. Briefly, 3 × 10^4^ cells were plated in the upper chamber in stem cell medium without growth factors. Stem cell medium supplemented with growth factors (EGF and bFGF) was added in the lower chamber and used as chemoattractant. After 48h, the fluorescent dye calcein acetoxymethyl ester (calcein-AM, Life Technologies Corporation) was added to the lower chamber and incubated for 30 min at 37 °C. The cell viability indicator, calcein-AM, is a non-fluorescent, cell permeate compound that is hydrolyzed by intracellular esterases into the fluorescent anion calcein and can be used to fluorescently label viable cells before microscope observation. The number of migrated cells was evaluated by counting the cells after imaging acquisition using a fluorescence microscope.

Colony formation ability of transduced GSCs was evaluated by plating a single cell/well in 96-well plates. After 3-4 weeks, each well was examined and the number of spheres/cell aggregates were counted. For TRIPZ and TRIPZ-miR-370 GSCs, all the experiments were performed in stem cell medium in the presence of doxycycline.

### 4.6. Orthotopic Xenograft Mouse Models

Animal experiments were performed in accordance to relevant institutional and national regulations. Moreover, 2 × 10^5^ TRIPZ and TRIPZ-miR-370GSCs were intracranially injected into NOD/SCID mice (*n, 6*; 4–6 weeks of age; CD1 NOD-/SCID mice, Charles Rives, Italy). Before grafting, mice were anesthetized with intraperitoneal injection of diazepam (2 mg/100 g) followed by intramuscular injection of ketamine (4 mg/100 g). The animal skulls were immobilized in a stereotactic head frame and a burr hole was made 2 mm right of the midline and 1 mm posterior to the coronal suture. The tip of a 10-µl Hamilton micro-syringe was placed at a depth of 3 mm from the dura and the cells were slowly injected. Doxycycline administration in drinking water (200 µg/mL) started the day of injection. After 4 weeks of survival, mice were deeply anesthetized and transcardially perfused with 0.1 M PBS (pH = 7.4), followed by 4% paraformaldehyde in 0.1 M PBS. The brain was removed, stored in 30% sucrose buffer overnight at 4° C and serially cryotomed at 20 µm on the coronal plane. Sections were collected in distilled water, mounted on slides, and cover-slipped with Eukitt. Images were obtained with a Laser Scanning Confocal Microscope (IX81, Olympus Inc., Melville, NY, USA).

Tumor volumes were calculated on histological section through the tumor epicenter, according to the Equation (1),

(1) $$V=(a^{2}\times b)/2$$

where *a* is the shortest diameter and *b* is the longest diameter of tumors.

Immunofluorescence was performed as previously described [71], utilizing antiserum directed against Ki67 (Dako, Glostrup, Denmark). To calculate the percentage of antigen-expressing tumor cells, at least 1000 cells were counted across 10 different fields.

### 4.7. Noncoding-RNA Target Prediction

TargetScan (http://www.targetscan.org), miRanda (http://www.microrna.org), starBase v2.0 (http://starbase.sysu.edu.cn) were used for miR-370-3p target prediction.

### 4.8. Reporter Assay

The human HIF1A 3′-UTR and NEAT1 sequences containing the target sites for miR-370-3p were amplified by PCR from genomic DNA and cloned into pGL3 control vector or psiCHECK(TM)-2 Vector (Promega Inc.) downstream of the luciferase reporter gene. The mutant plasmid containing mutations of the miR-370-3p target sites were obtained by site-specific mutagenesis. All the plasmids were verified by sequence analysis.

Human 293T cells were transiently co-transfected by Lipofectamine 2000 (Invitrogen), with 400 ng of luciferase reporter plasmid containing wild-type or mutated NEAT1-seed or HIF1A 3′-UTR-seed sequences for miR-370-3p, 10 pmol of either the hsa-miR370-3p mimic or control-mimic oligonucleotides (Ambion, Life technologies). Thirty-six h post-transfection luciferase activity was quantified by Dual Luciferase Reporter kit (Promega Inc.)

### 4.9. Gene Array

Gene array was performed as previously described [72]. Briefly, total RNA was extracted from TRIPZ and TRIPZ-miR-370 GSC#1 transduced cells. RNA was labeled and hybridized to the Affymetrix GeneChip1.0 ST array (Affymetrix, Santa Clara, CA, USA) following the manufacturer′s instructions. Hybridization values were normalized by the RMA method.

### 4.10. Statistical Analysis

Statistical analysis was performed by means of GraphPad prism v4.0 (GraphPad Software, La Jolla, CA, USA, www.graphpad.com). Statistical significance reported on the plots is the following: * *p* < 0.05, ** *p* < 0.01 and *** *p* < 0.001.

## Figures and Tables

**Table 1:** List of primers.

	For: 5′–>3′	Rev: 5′–>3′
**NEAT1**	TGCTTGTTCCAGAGCCCATGAATGCCA	GTTCTACAGCTTAGGGATCTTCTTGAAGC
**HIF1A**	CATCAGCTATTTGCGTGTGAGGA	AGCAATTCATCTGTGCTTTCATGTC
**HMGA2**	AGTCCCTCTAAAGCAGCTCA	GTCCTCTTCGGCAGACTCTT
**GAPDH**	ACCTGACCTGCCGTCTAGAAAA	CCTGCTTCACCACCTTCTTGA

## Supplementary Materials

Supplementary materials can be found at https://www.mdpi.com/1422-0067/21/10/3610/s1.
