# Supplementary material for: Mir-370-3p Impairs Glioblastoma Stem-Like Cell Malignancy Regulating a Complex Interplay between HMGA2/HIF1A and the Oncogenic Long Non-Coding RNA (lncRNA) NEAT1
Source: Int J Mol Sci. 2020 May 20;21(10):3610. doi: 10.3390/ijms21103610 (PMC7279259; doi:10.3390/ijms21103610)
Supplement: Supplementary file 1 [file ijms-21-03610-s001.pdf]

Supplementary Table 1. Clinical and pathological features of Glioblastoma patients.

Clinical features	Data
**Median Age (range)**	57 (42-72)
**M/F**	21/6
**Localization**	
Parietal	7
Frontal	11
Occipital	2
Temporal	7
**Median Karnofsky performance (range)**	75 (40-90)
**Survival median (range)**	12.2 (2-42)
**MGMT status (M/UM)**	13/14

Supplementary Table 2: Overlap of modulated genes in GSC#1 cells with restored expression of miR-370. Hallmark gene sets with GSEA are shown**.**

Entrez Gene Id	Gene Symbol	HALLMARK EPITHELIAL MESENCHYMAL TRANSITION	HALLMARK TNFA SIGNALING VIA NFKB	HALLMARK UV RESPONSE DN	HALLMARK HEDGEHOG SIGNALING	HALLMARK HYPOXIA	HALLMARK IL2 STAT5 SIGNALING	HALLMARK INFLAMMATORY RESPONSE	HALLMARK INTERFERON GAMMA RESPONSE	HALLMARK KRAS SIGNALING UP	HALLMARK COAGULATION
7422	**VEGFA**	■	■		■	■					
7128	**TNFAIP3**	■	■			■			■	■	
56937	**PMEPA1**	■	■								
2669	**GEM**	■	■								
3486	**IGFBP3**	■				■				■	
4907	**NT5E**	■					■				
333	**APLP1**	■					■				
4323	**MMP14**	■						■			■
5396	**PRRX1**	■								■	
4313	**MMP2**	■									■
7057	**THBS1**	■									■
5352	**PLOD2**	■			■						
7040	**TGFB1**	■									
51330	**TNFRSF12A**	■									
22795	**NID2**	■									
5744	**PTHLH**	■									
2152	**F3**		■	■		■		■			■
2353	**FOS**		■			■					
2643	**GCH1**		■					■	■		
6347	**CCL2**		■					■	■		
7130	**TNFAIP6**		■					■	■		
2150	**F2RL1**		■							■	
8878	**SQSTM1**		■								
5606	**MAP2K3**		■								
1960	**EGR3**		■								
4929	**NR4A2**		■								
5271	**SERPINB8**		■								
57018	**CCNL1**		■								
7436	**VLDLR**			■	■	■					
8829	**NRP1**			■	■		■			■	
4233	**MET**			■				■			
3037	**HAS2**			■				■			
7035	**TFPI**			■						■	
5577	**PRKAR2B**			■							
23266	**ADGRL2**			■							
23345	**SYNE1**			■							
23576	**DDAH1**			■							
6334	**SCN8A**			■							
7048	**TGFBR2**			■							
1400	**CRMP1**				■						
154796	**AMOT**				■						
8828	**NRP2**				■						
10397	**NDRG1**					■	■				
5155	**PDGFB**					■					■
6781	**STC1**					■					
54206	**ERRFI1**					■					
8406	**SRPX**					■					

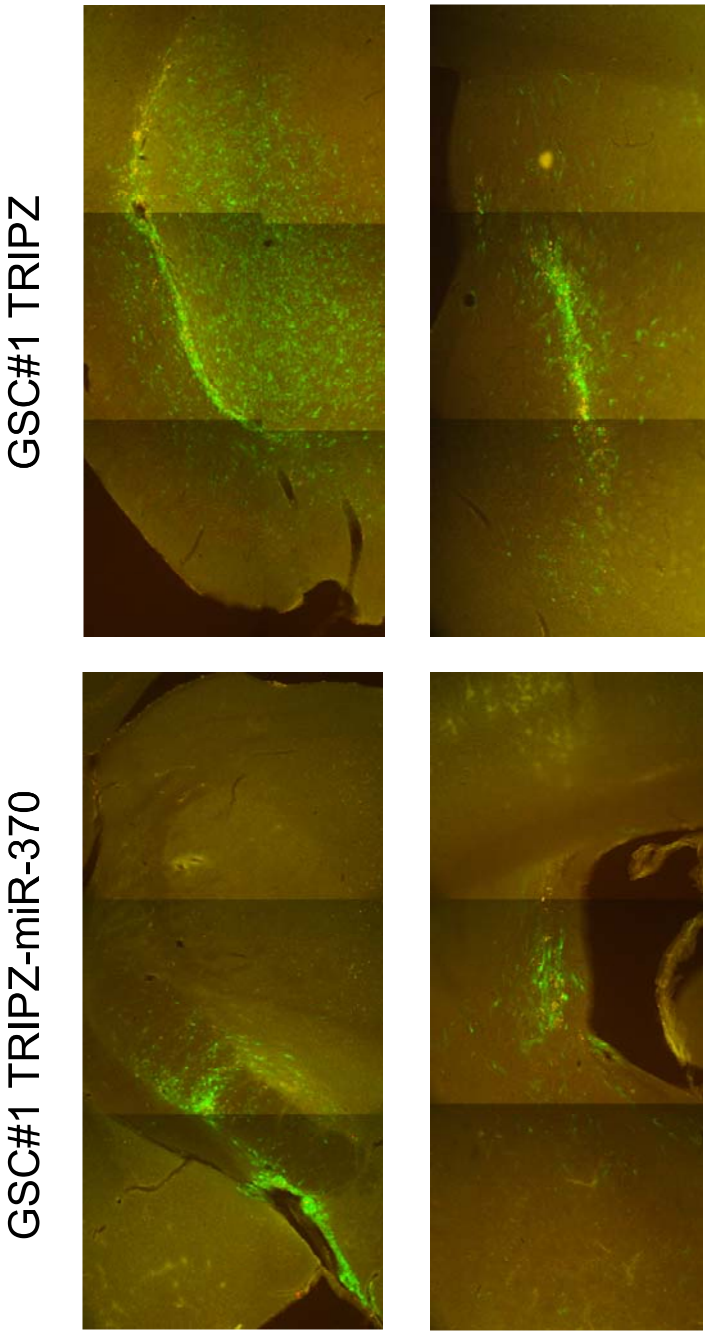

Supplementary Figure S1. Fluorescence microscopy of mouse brain grafted onto the right striatum with GFP+ GSC#1 cells (*green*). Photomontage of coronal section in a TRIPZ xenograft (*upper panel*) and in a TRIPZ-miR-370 xenograft *(lower panel*) showing the injection area. Scale bars, 200 μm.

284119	**CAVIN1**										
25819	**NOCT**										
55076	**TMEM45A**										
7133	**TNFRSF1B**										
4860	**PNP**										
115361	**GBP4**										
214	**ALCAM**										
990	**CDC6**										
4839	**NOP2**										
11230	**PRAF2**										
4135	**MAP6**										
5269	**SERPINB6**										
6335	**SCN9A**										
3091	**HIF1A**										
684	**BST2**										
6376	**CX3CL1**										
10630	**PDPN**										
8671	**SLC4A4**										
10437	**IFI30**										
51015	**ISOC1**										
196410	**METTL7B**										
25939	**SAMHD1**										
8869	**ST3GAL5**										
8404	**SPARCL1**										
23531	**MMD**										
23643	**LY96**										
26499	**PLEK2**										
3018	**HIST1H2BB**										
5801	**PTPRR**										
2934	**GSN**										
2	**A2M**										
11098	**PRSS23**										
1515	**CTSV**										
4485	**MST1**										
